# Radiofrequency ablation via an implanted self-expandable metallic stent to treat in-stent restenosis in a rat gastric outlet obstruction model

**DOI:** 10.3389/fbioe.2023.1244569

**Published:** 2023-09-06

**Authors:** Dong-Sung Won, Yubeen Park, Chu Hui Zeng, Dae Sung Ryu, Ji Won Kim, Jeon Min Kang, Song Hee Kim, Hyung-Sik Kim, Sang Soo Lee, Jung-Hoon Park

**Affiliations:** ^1^ Biomedical Engineering Research Center, Asan Institute for Life Sciences, Asan Medical Center, Seoul, Republic of Korea; ^2^ Department of Gastroenterology, Asan Medical Center, University of Ulsan College of Medicine, Seoul, Republic of Korea; ^3^ Department of Mechatronics Engineering, School of ICT Convergence Engineering, College of Science and Technology, Konkuk University, Chungju, Republic of Korea

**Keywords:** radiofrequency ablation, self-expandable metallic stent, gastric outlet obstruction, tissue hyperplasia, granulation tissue

## Abstract

**Background:** In-stent restenosis caused by tissue hyperplasia and tumor growth through the wire meshes of an implanted self-expandable metallic stent (SEMS) remains an unresolved obstacle. This study aimed to investigate the safety and efficacy of SEMS-mediated radiofrequency ablation (RFA) for treating stent-induced tissue hyperplasia in a rat gastric outlet obstruction model.

**Methods:** The ablation zone was investigated using extracted porcine liver according to the ablation time. The optimal RFA parameters were evaluated in the dissected rat gastric outlet. We allocated 40 male rats to four groups of 10 rats as follows: group A, SEMS placement only; group B, SEMS-mediated RFA at 4 weeks; group C, SEMS-mediated RFA at 4 weeks and housed until 8 weeks; and group D, SEMS-mediated RFA at 4 and 8 weeks. Endoscopy and fluoroscopy for *in vivo* imaging and histological and immunohistochemical analysis were performed to compare experimental groups.

**Results:** Stent placement and SEMS-mediated RFA with an optimized RFA parameter were technically successful in all groups. Granulation tissue formation-related variables were significantly higher in group A than in groups B–D (all *p* < 0.05). Endoscopic and histological findings confirmed that the degrees of stent-induced tissue hyperplasia in group D were significantly lower than in groups B and C (all *p* < 0.05). Hsp70 and TUNEL expressions were significantly higher in groups B–D than in group A (all *p* < 0.001).

**Conclusion:** The implanted SEMS-mediated RFA successfully managed stent-induced tissue hyperplasia, and repeated or periodic RFA seems to be more effective in treating in-stent restenosis in a rat gastric outlet obstruction model.

## Introduction

Endoscopic or fluoroscopic placement of a self-expandable metallic stent (SEMS) has been considered a standard therapeutic option for palliating unresectable malignant gastric outlet obstruction (GOO) (Park et al., 2016a; Park et al., 2016b). Stent placement is an effective modality associated with faster symptom relief and shorter hospital stay, and it is cheaper than surgical management (Dormann et al., 2004; Jeurnink et al., 2010; Park et al., 2016b). However, a high risk of progressive tissue ingrowth or overgrowth through the stent meshes has been reported, and these situations are still considered significant obstacles to successful stent placement (Wadhwa et al., 2003; Kim et al., 2014; Jeong, 2016). To treat tissue hyperplasia or tumor growth adjacent to the implanted SEMS in non-vascular luminal organs, various functionalized stents with the use of antiproliferative or anticancer drugs, small interfering RNA, nanomaterials, and shape modifications have been developed and investigated (Lee et al., 2009; Van Hooft et al., 2011; Kim et al., 2014; Park et al., 2014; Jun et al., 2017; Jeon et al., 2022; Park et al., 2022). However, it remains an unresolved problem in the clinic, and a standard therapeutic strategy has not been established (Park et al., 2019).

Controlled photothermal therapy using nanofunctionalized stents effectively prevented and/or treated in-stent restenosis caused by tissue hyperplasia after stent placement in various animal models (Park et al., 2018; Cho et al., 2021; Hu et al., 2021). Furthermore, radiofrequency ablation (RFA) via an endobiliary RF catheter successfully treated the occluded SEMS in patients with a malignant biliary obstruction (Mizandari et al., 2018; Auriemma et al., 2019; Larghi et al., 2019). The application of RFA prolonged stent patency by inducing coagulative necrosis in the proliferated tissues (Kadayifci et al., 2016). The passage of a guide wire with RF electrodes into the occluded SEMS is required to apply RFA. In the case of high-level progressive tumor growth with severe strictures and tortuous anatomical variables, technical difficulties might occur (Jang et al., 2017; Paik et al., 2020). Therefore, we hypothesized that SEMS-mediated RFA using a monopolar SB Knife would be able to grasp the implanted SEMS to treat stent-induced granulation tissue developed after SEMS placement. This study aimed to investigate the safety and efficacy of SEMS-mediated RFA for treating stent-induced tissue hyperplasia after SEMS placement in a rat GOO model.

## Methods

### Stent construction and the RFA system

The SEMS was knitted with a single 0.127-mm nitinol wire thread (S&G biotech, Yongin, Korea). At full expansion, the stent was 5 mm in diameter and 10 mm in length. A 6-Fr sheath (Cook, Bloomington, IN, United States) and a pusher catheter were used to deliver the SEMS.

The monopolar SB Knife Jr W-type (Sumitomo Bakelite Co., Ltd., Kanagawa, Japan) was used to deliver RF energy to the implanted SEMS from the CoATherm AK-F200 RF generator (APRO KOREA, Gunpo, Korea). The power output range of the RF generator was 5–200 W, with a fixed frequency of 480 kHz.

### Ablation ranges of SEMS-mediated RFA in the porcine liver

The ablation ranges of SEMS-mediated RFA were investigated using the extracted porcine liver (BIOZOA biological, Seoul, Korea). A 4-mm-wide cylindrical stainless-steel straw was used to create a cylindrical hole in the midline of the porcine liver. The SEMS was precisely positioned into the cylindrical hole of the porcine liver under fluoroscopic guidance. The stented liver was placed on a ground pad, and the proximal end of the implanted SEMS was grasped with the monopolar SB knife. The five different RFA times of 60, 120, 180, 240, and 300 s were investigated and repeated five times to ensure statistical reproducibility. The baseline RFA parameter was set at 60°C, 15 W, and 480 kHz. The power was controlled at a 3-s interval to maintain the temperature at 60°C. A thermal camera (FLIR A400; Teledyne FLIR, Wilsonville, OR, United States) was used to examine the temperature and thermal images of SEMS during the procedure. The ablated liver was sectioned along the placed SEMS axis. A ruler was used to measure the ablated depth and length of each sample at eight different points.

### Animal study design

The Institutional Animal Care and Use Committee (grant noo. 2021-14-231) approved the experimental protocol, and the animals used in this study are in accordance with the United States National Institutes of Health guidelines for the humane handling of laboratory animals.

A total of 44 male Sprague–Dawley rats (weighing, 215–275 g; JA BIO, Suwon, Korea) were used in the study. Four rats were used to determine the optimization of the RFA parameter, and the remaining 40 rats were used to investigate the safety and efficacy of SEMS-mediated RFA. The 40 rats were randomly divided into four groups after SEMS placement: group A (*n* = 10) received SEMS placement only and was housed for 4 weeks; group B (*n* = 10) received SEMS-mediated RFA at 4 weeks; group C (*n* = 10) received SEMS-mediated RFA at 4 weeks and was housed for 8 weeks; and group D (*n* = 10) received SEMS-mediated RFA both at 4 and 8 weeks. Rats in groups A and B were sacrificed at 4 weeks, while the remaining rats in groups C and D were sacrificed at 8 weeks to evaluate the rebound effects of RFA. All animals intramuscularly received 1 mg/kg of Keromin (Hana Pharm, Seoul, Korea) for pain control and 0.08 mg/kg gentamicin (Shin Poong Pharm, Seoul, Korea) for 3 days after the procedure. Each rat was weighed weekly until euthanization. All animals were housed under a 12-h control cycle at 55% ± 10% relative humidity and at 24°C ± 1°C environmental temperature. All animals were euthanized by administering inhalable pure carbon dioxide at the corresponding time.

### Optimization of the RFA parameter in the rat gastric outlet

The SEMS-mediated RFA was performed in the surgically exposed rat gastric outlet to determine the optimal RFA parameter. The RFA was performed at 15 W, 60°C, and 480 kHz and for 120, 180, 240, and 300 s. A thermal camera (FLIR A400) was used to obtain the temperature and thermal images during the procedure. Gross and histological examinations, including hematoxylin and eosin (H&E) staining and immunohistochemistry (IHC) with heat shock protein 70 (Hsp70; LS-B3700-50; LifeSpan BioSciences Inc., Washington, SA, United States) and terminal deoxynucleotidyl transferase-mediated dUTP nick and labeling (TUNEL; ApopTag Peroxidase *In Situ* Detection Kit; Millipore Co., Burlington, MA, United States), were performed to evaluate the tissue changes after SEMS-mediated RFA in the rat GOO model. After the procedure, all rats were immediately euthanized using inhalable pure carbon dioxide.

### SEMS placement in the rat gastric outlet

The stent placement techniques have been described in detail previously (Park et al., 2020). In brief, a 1.5-cm midline incision was made after anesthesia under sterile conditions. A 0.5-mm-long incision was made on the stomach’s anterior wall. The sheath with a loaded SEMS was inserted into the gastric outlet through the incised stomach under fluoroscopic guidance. The SEMS were carefully placed into the gastric outlet by pulling the sheath while maintaining the pusher catheter in place. The proximal end of the SEMS was sutured to the gastric outlet tissue to prevent migration. All incision sites were closed with sutures, including the stomach, peritoneum, and skin.

### SEMS-mediated RFA procedure

SEMS-mediated RFA was performed at 4 weeks in groups B, C, and D and at 8 weeks in group D. The aforementioned SEMS placement technique was repeated to reach the implanted SEMS. A 0.5-cm-long incision of the stomach was made around the proximal end of the implanted SEMS. The monopolar SB knife was inserted into the incision site and grasped the proximal end of the implanted SEMS under fluoroscopic guidance. SEMS-mediated RFA was used for 180 s at 15 W while maintaining a temperature of 60°C. A thermal camera (FLIR A400) obtained temperature changes and thermal images during the procedure.

### Follow-up endoscopic and fluoroscopic examinations

The endoscopic examination was performed to evaluate the mucosal changes of the ablated gastric outlet before and immediately after SEMS placement, before and immediately after the RFA procedure, and before being euthanized at 4 weeks and 8 weeks. The endoscope (CMOS Video-Rhino-Laryngoscope, KARL STORZ, Tuttlingen, Germany) was carefully inserted through the incision site of the stomach. The degree of stent-induced tissue hyperplasia was subjectively graded using the obtained endoscopic images as follows: 1, none; 2, mild (covering up to 25% of the stent circumference); 3, moderate (covering 50% of the stent circumference); and 4, severe (covering more than 50% of the stent circumference) (Becker et al., 2005; Koelink et al., 2018). The endoscopic findings were analyzed using the consensus of three observers blinded to the study.

The follow-up contrast study was performed immediately after SEMS placement, before and after RFA procedure, and before being euthanized to check stent position, patency, and the presence of perforation using a contrast medium (Telebrix Gastro; Guerbet, Villepinte, France).

### Histological analysis

A gross examination of tissue changes was performed after the surgical exploration of the stented gastric outlet. The harvested tissue samples were fixed in 10% neutral buffered formalin for 48 h. The transverse sections of the samples were made and embedded in a paraffin block for H&E and Masson’s trichrome (MT) staining. H&E was used to evaluate the thickness of the submucosal fibrosis from the stent strut to the submucosal layer, and the percentage of tissue hyperplasia in the cross-sectional area of the gastric outlet was evaluated using the following formula: 100 × [1 − (stenotic area of stent/original area of stent)] (Park et al., 2018; Cho et al., 2021). On MT-stained slices, the degree of collagen deposition was subjectively determined as follows: 1, mild; 2, mild to moderate; 3, moderate; 4, moderate to severe; and 5, severe. The samples were histologically analyzed via a digital slide scanner (Pannoramic 250 FLASH III; 3D HISTECH Ltd., Budapest, Hungary), and measurements were taken using a digital microscope viewer (Supplementary Figure S1, CaseViewer; 3D HISTECH). The histological evaluations were determined by the agreement of three observers blinded to the experimental groups.

### Immunohistochemistry

IHC analysis was performed on paraffin-embedded sections using Hsp70, TUNEL, and α-smooth muscle actin (α-SMA; Abcam, Cambridge, England). The extents of Hsp70-, TUNEL-, and α-SMA-positive deposition were subjectively determined according to a previous study (1, mild; 2, mild to moderate; 3, moderate; 4, moderate to severe; and 5, severe) (Park et al., 2022).

### Statistical analysis

Data were expressed as mean ± standard deviation (SD). The Kruskal–Wallis or Mann–Whitney U test was used to analyze differences between the groups. A *p*-value <0.05 was considered statistically significant. SPSS software (version 27.0; SPSS, IBM, Chicago, IL, United States) was used for all statistical analyses.

## Results

### Ablation ranges of SEMS-mediated RFA in the porcine liver

**FIGURE 1:**
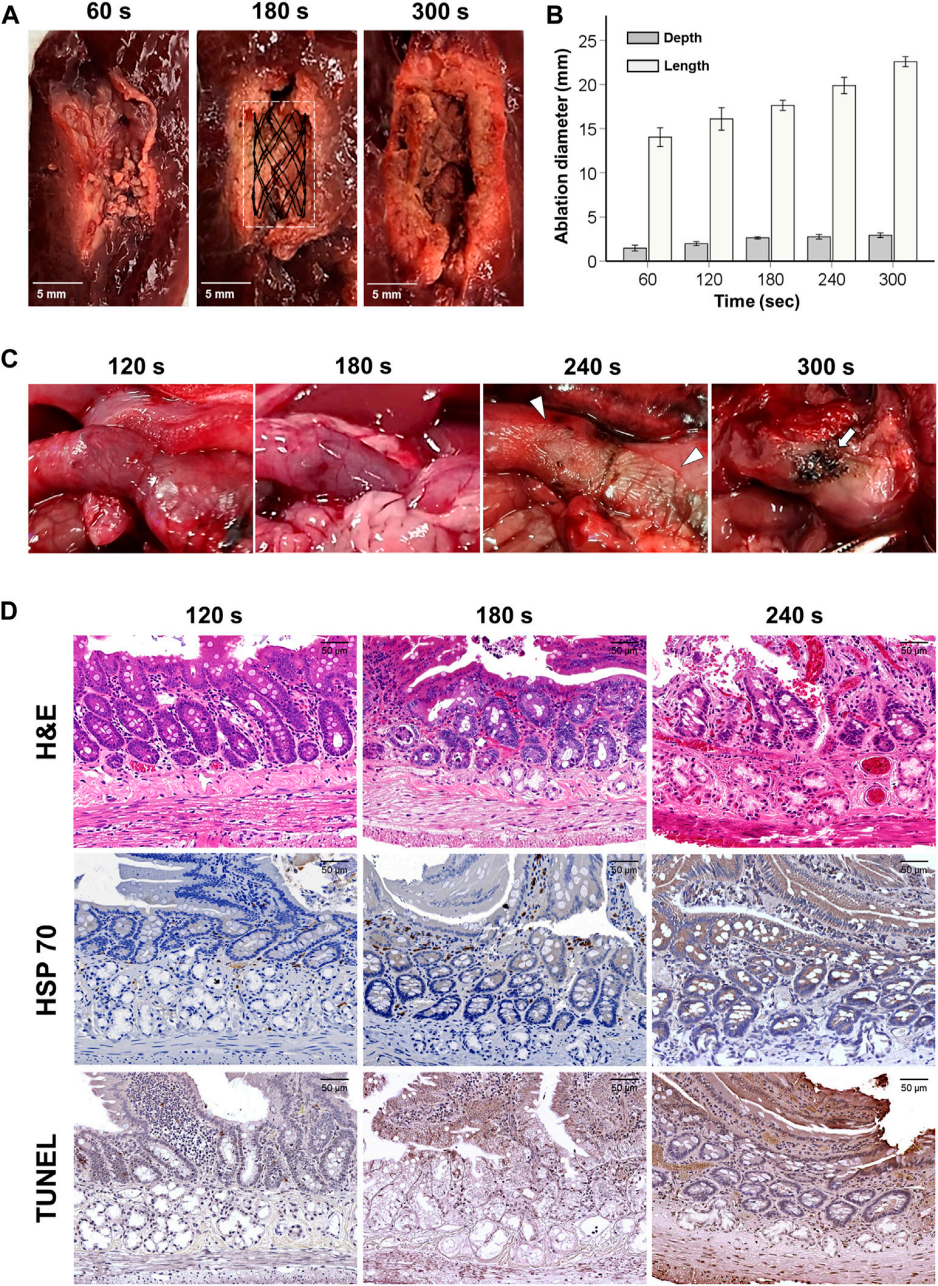
Gross, histological, and immunohistochemical findings immediately after radiofrequency ablation. **(A)** Representative images of the ablated liver at 60, 180, and 300 s. **(B)** Graph showing the ablation depth and length in the porcine liver at 60, 120, 180, 240, and 300 s. **(C)** Gross findings present tissue damages by various RFA time in the rat gastric outlet over time. Eventually, overheating caused tissue burning (*arrowheads*) at 240 s and tanned tissue (*arrows*) at 300 s. **(D)** As the RFA parameter increased, the heated reaction was confirmed along with cellular necrosis over the endothelial villi, intestinal glands, and duodenal glands.

The ablation ranges of SEMS-mediated RFA are shown in Figures 1A, B. SEMS-mediated RFA in all RF parameters was successfully performed in the extracted porcine liver. However, RFA at 60 s failed to create a uniform circumferential shape. A uniform circumferential ablation zone in the remaining groups was successfully created. The ablation depth and length increased proportionally to the time. The mean (±SD) ablation depth was 1.47 ± 0.52 mm at 60 s, 1.97 ± 0.34 mm at 120 s, 2.63 ± 0.19 mm at 180 s, 2.75 ± 0.40 mm at 240 s, and 2.93 ± 0.41 mm at 300 s. The mean ablation length was 14.04 ± 1.70 mm at 60 s, 16.10 ± 2.02 mm at 120 s, 17.63 ± 0.92 mm at 180 s, 19.89 ± 1.46 mm at 240 s, and 22.58 ± 0.88 mm at 300 s.

### Optimal RFA parameters in the rat gastric outlet

SEMS-mediated RFA was performed successfully in all rats. Mucosal injuries with thermal damages around the implanted SEMS were observed at 120 and 180 s, while severe mucosal injuries with color changes in the gastric outlet tissues were observed at 240 s. The gastric outlet was tanned at 300 s, and severe mucosal injuries with ductal perforation were observed in the stented gastric outlet (Figure 1C). Mild Hsp70- and TUNEL-positive depositions were consistently observed around the villi at 120 s in histological findings. At 180 s, there was a moderate Hsp70- and TUNEL-positive deposition across the pyloric glands. However, at 240 s, overheated damages and hyper-cellular apoptosis were observed throughout the tissue. Histological examinations could not be performed at 300 s due to severe mucosal injuries with perforation (Figure 1D). Based on the gross and histological results, the optimal RF time was chosen as 180 s.

### Procedural outcomes of the implanted SEMS-mediated RFA in the rat gastric outlet

**FIGURE 2:**
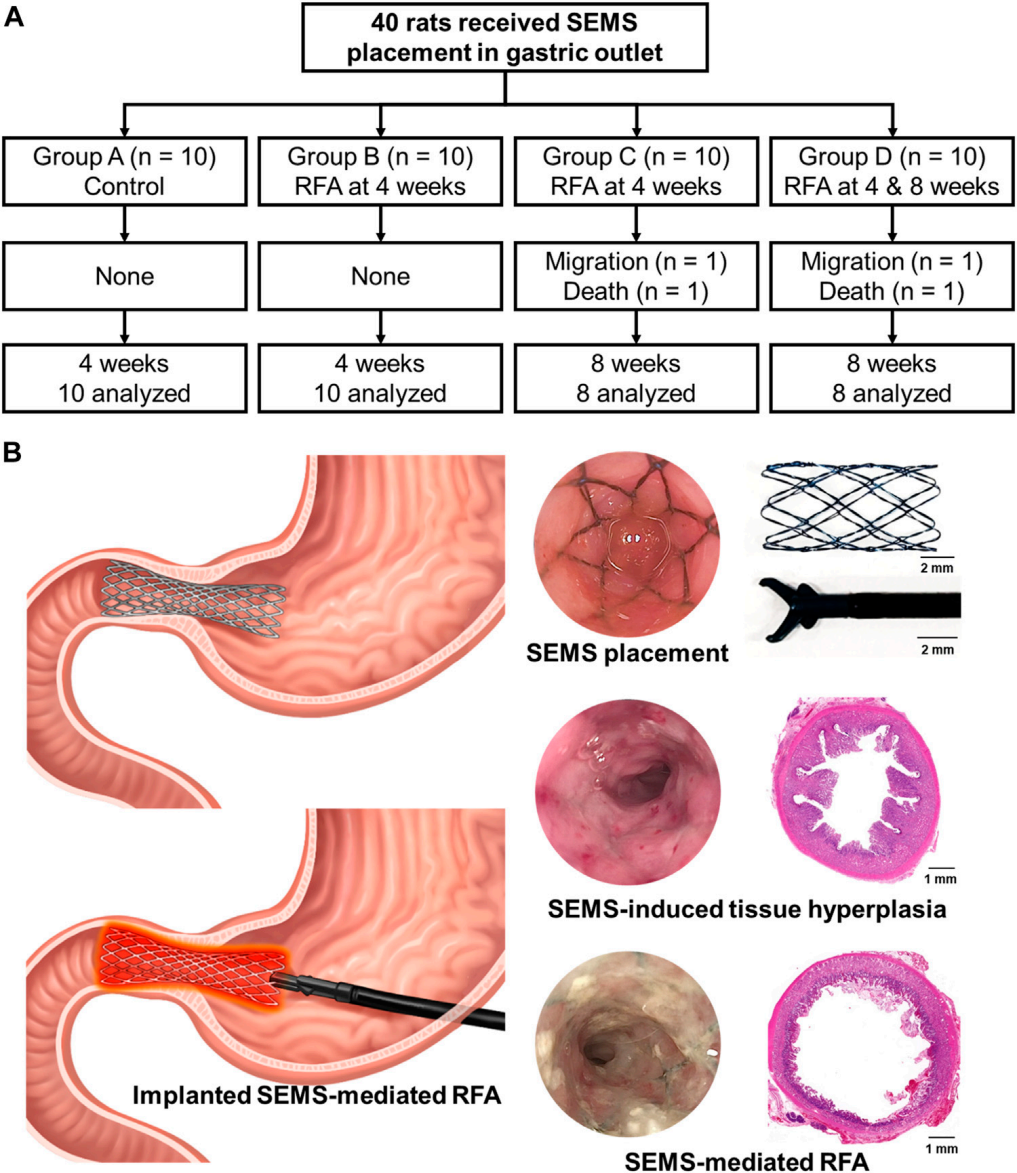
Study design for an *in vivo* experiment. **(A)** Flowchart showing the randomized study process and follow-up. **(B)** Schematic illustration of radiofrequency ablation via an implanted self-expandable metallic stent in the rat gastric outlet obstruction model to treat in-stent restenosis.

**FIGURE 3:**
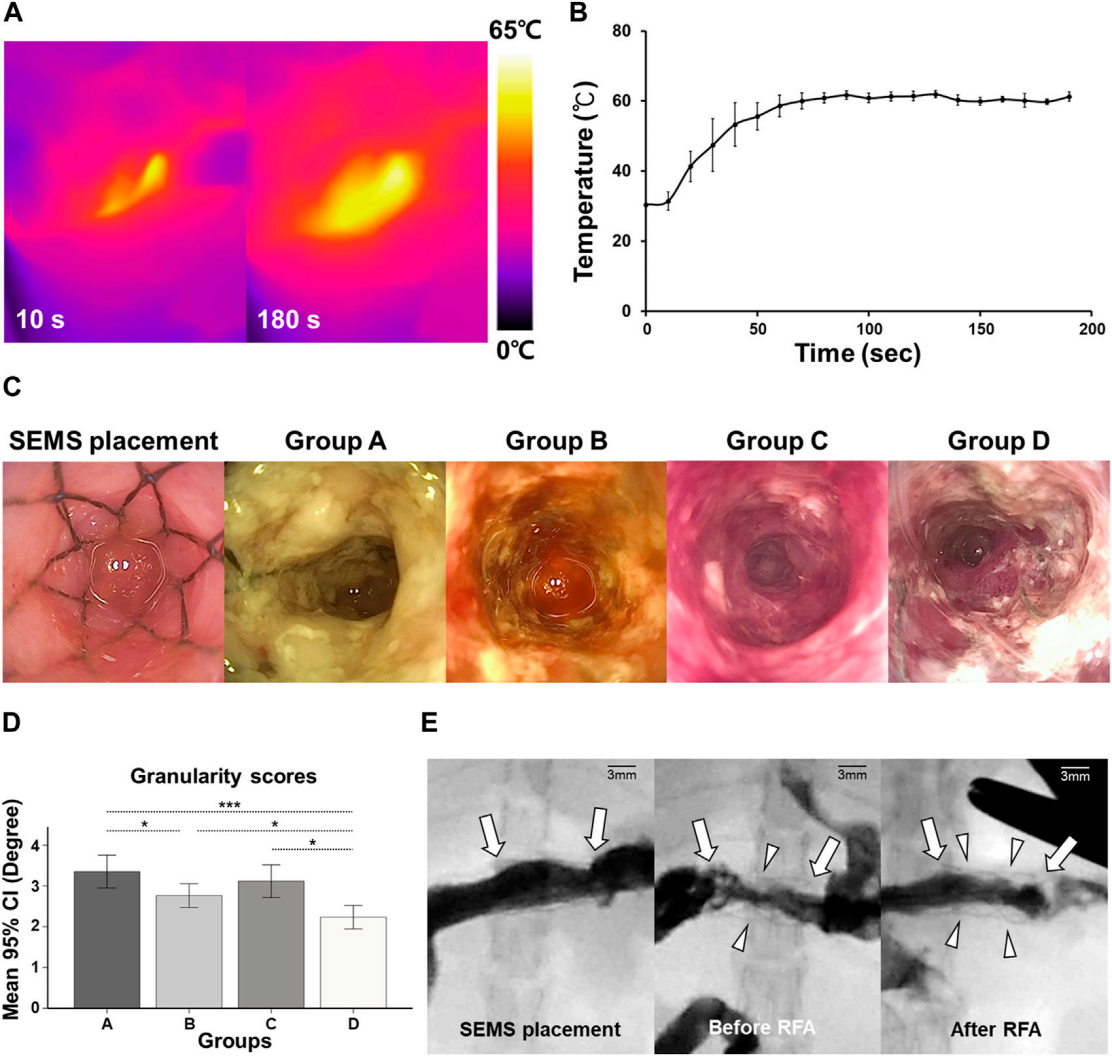
*In vivo* experimental imaging findings. **(A)** Representative thermal images obtained during the radiofrequency ablation at 10 and 180 s, 60°C, 15 W, and 480 kHz. **(B)** Graph showing the temperature changes during self-expandable metallic stent-mediated RFA. **(C)** Representative endoscopic images showing mucosal changes before being euthanized in each group. **(D)** Graph of the endoscopic finding indicating a change of granularity scores. **(E)** Representative fluoroscopic images obtained during the follow-up study showed the implanted SEMS (arrows) immediately after SEMS placement and stent patency (arrowheads) before and after SEMS-mediated RFA.

SEMS placement and SEMS-mediated RFA were technically successful. However, two rats from groups C and D died within 3 days after the RFA procedure due to a post-operative sepsis. Stent migration occurred in two rats from groups C and D at a 4-week follow-up. These four rats were excluded from the study. The remaining 36 rats survived until the end of the study without additional complications (Figure 2). The temperature during the procedure at 15 W and 480 kHz was gradually increased up to 60°C within 70 s. The mean steady-state temperature was 60.75°C ± 1.86°C (Figures 3A, B).

The body weight of the surviving rats decreased in the first week after the RFA procedure, but this had no effect on their behavior or quality of life, and they eventually recovered. Additionally, there was no significant difference in the body weight among the groups after SEMS placement and RFA procedure (Supplementary Figure S2).

### Endoscopic and fluoroscopic findings

The representative endoscopic images before euthanizing in study groups are shown in Figure 3C. Endoscopic images revealed severe in-stent stenosis caused by stent-induced tissue hyperplasia through the stent meshes in group A. Stent patency in group B was relatively improved after SEMS-mediated RFA compared with group A. However, at the 8-week follow-up, the rebound effects after RFA were observed in group C. Endoscopic images obtained after repeated SEMS-mediated RFA in group D revealed the successfully maintained stent patency. The mean granularity grade scores differed significantly between the groups (all variables; *p* < 0.001, Kruskal–Wallis test). The mean granularity grade scores were significantly lower in group D (2.23 ± 0.56) than in groups A (3.35 ± 0.78; *p* < 0.001), B (2.76 ± 0.56; *p* < 0.05), and C (3.11 ± 0.78; *p* < 0.05). Moreover, the mean granularity grade scores were significantly lower in group B than in group A (*p* < 0.05) However, there was no significant difference in the mean granularity grade scores between group C and groups A and B (all variables; *p* > 0.05) (Figure 3D).

In the fluoroscopic findings, good passage of the contrast medium was observed immediately after SEMS placement in all rats. However, moderate and severe luminal narrowing was observed throughout the SEMS at 4 weeks after SEMS placement in group A. There was no evidence of perforation, and the luminal patency was slightly wider immediately after the SEMS-mediated RFA procedure in groups B, C, and D (Figure 3E).

### Histological findings

**FIGURE 4:**
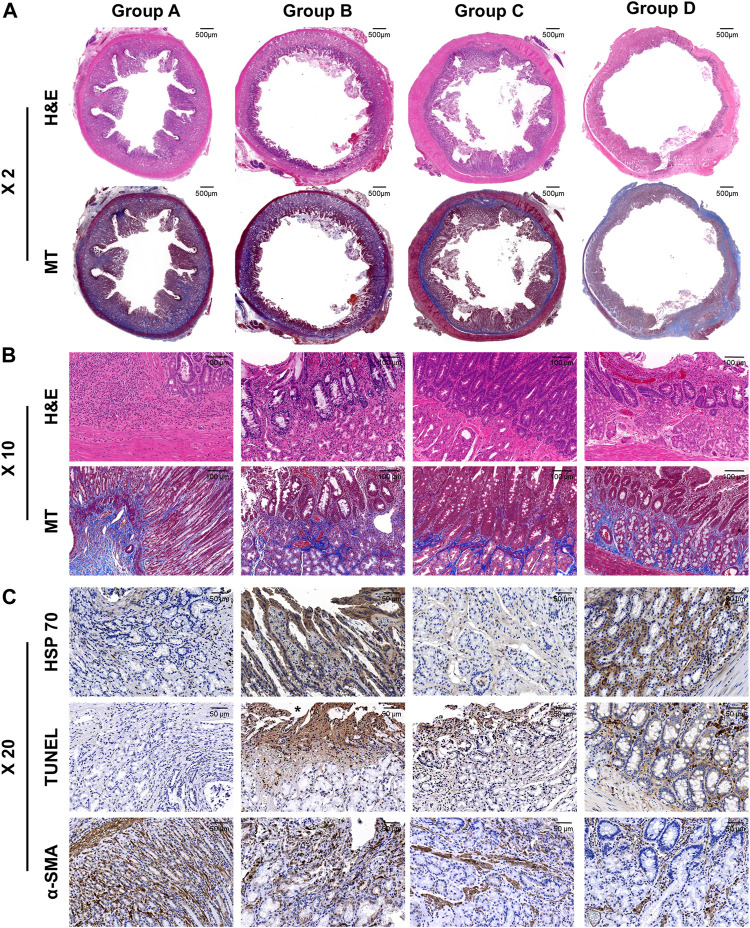
Representative histological and immunohistochemical images of hematoxylin and eosin, Masson’s trichrome, heat shock protein 70, terminal deoxynucleotidyl transferase mediated dUTP nick and labeling, and α-smooth muscle actin of all the groups. **(A)** ×2 magnification and **(B)**×10 magnification of H&E and MT slices are showing radiofrequency-ablated tissue changes serial groups. **(C)** X20 IHC images are showing cellular apoptosis and regenerative reaction.

**FIGURE 5:**
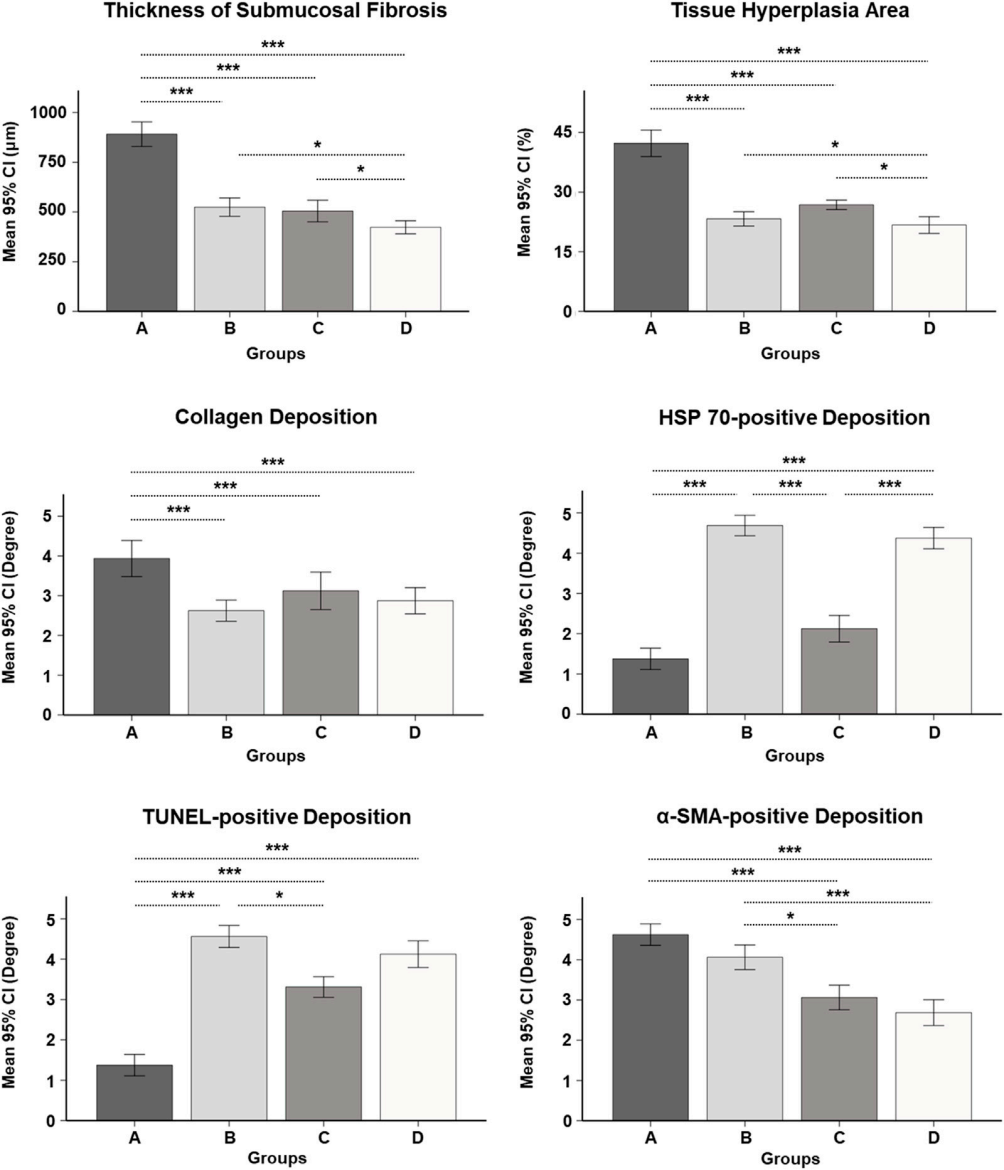
Serial histopathological findings for the gastric outlet obstruction model at 4 and 8 weeks after self-expandable metallic stent placement with/without SEMS-mediated radiofrequency ablation in groups A, B, C, and D. * <0.05, *** <0.001.

The histologic findings are summarized in Table 1 and shown in Figure 4. The mean thickness of submucosal fibrosis, percentages of tissue hyperplasia area, and collagen deposition were all significantly different between the groups (all variables; *p* < 0.001, Kruskal–Wallis test). The mean thickness of submucosal fibrosis, percentages of tissue hyperplasia area, and collagen deposition in groups B, C, and D were significantly lower than those in group A (all variables; *p* < 0.05). The mean thickness of submucosal fibrosis and tissue hyperplasia area was significantly lower in group D than in groups B and C (all variables; *p* < 0.05). However, there was no significant difference in the mean thickness of submucosal fibrosis and tissue hyperplasia area (all variables; *p* > 0.05) between groups B and C. Additionally, there was no significant difference in mean collagen deposition among groups B, C, and D (all variables; *p* > 0.05) (Figure 5).

**TABLE 1:** *In vivo* experimental study findings after self-expandable metallic stent placement with or without radiofrequency ablation.

	Group A	Group B	Group C	Group D	^*^ *p*-value	^*+*^ *p*-value					
						A vs. B	A vs. C	A vs. D	B vs. C	B vs. D	C vs. D
Thickness of submucosal fibrosis (μm)	891.40 ± 115.82	524.74 ± 86.66	504.88 ± 102.11	423.32 ± 61.55	<0.001	0.001	0.001	0.001	0.683	0.008	0.014
Tissue hyperplasia area (%)	42.28 ± 6.24	24.17 ± 2.67	26.81 ± 2.21	21.73 ± 3.94	0.001	0.001	0.001	0.001	0.648	0.049	0.027
Collagen deposition (degree)	3.93 ± 0.85	2.62 ± 0.50	3.12 ± 0.88	2.87 ± 0.61	0.001	0.001	0.022	0.009	0.082	0.366	0.488
Hsp70 (degree)	1.40 ± 0.51	4.68 ± 0.47	2.12 ± 0.61	4.37 ± 0.50	0.001	0.001	0.138	0.001	0.001	0.781	0.001
TUNEL (degree)	1.37 ± 0.50	4.56 ± 0.51	3.31 ± 0.47	4.12 ± 0.61	0.001	0.001	0.001	0.001	0.004	0.912	0.154
α-SMA (degree)	4.62 ± 0.59	4.06 ± 0.57	3.13 ± 0.54	2.73 ± 0.60	0.001	0.646	0.001	0.001	0.010	0.001	0.846

Data are presented as mean ± standard deviation. *Kruskal–Wallis test + Mann–Whitney U test. Hsp70: heat shock protein 70, TUNEL: terminal deoxynucleotidyl transferase-mediated dUTP, and α-SMA: α-smooth muscle actin.

### Immunohistochemistry findings

The mean degrees of Hsp70-, TUNEL-, and α-SMA-positive depositions were significantly different among the groups (all variables; *p* < 0.001, Kruskal–Wallis test). The mean degrees of Hsp70-positive deposition in groups B and D were significantly higher than those in groups A and C (all variables; *p* < 0.001). However, there were no significant differences in the mean degrees of Hsp70-positive deposition between groups B and D (*p* = 0.781). The mean degrees of TUNEL-positive deposition were significantly higher in groups B, C, and D than those in group A (all variables; *p* < 0.001). Moreover, the mean degree of TUNEL-positive deposition was significantly higher in group B than that in group C (*p* < 0.05). However, there were no significant differences in the mean degrees of TUNEL-positive deposition between group D and groups B and C (all variables; *p* > 0.05). The mean degrees of α-SMA-positive deposition were significantly lower in groups C and D than those in groups A and B (all variables; *p* < 0.05). However, there were no significant differences in the mean degree of α-SMA-positive deposition between groups A and B and groups C and D (all variables; *p* > 0.05) (Figures 4, 5).

## Discussion

The results of the present study demonstrated that an implanted SEMS could successfully deliver RF energy to the target tissues via an endoscopic submucosal dissection knife, and the SEMS-mediated RFA effectively treated stent-induced tissue hyperplasia in the rat GOO model. Endoscopic and fluoroscopic findings confirmed that the RFA-treated groups had less in-stent restenosis and improved stent patency compared with the non-treated group. Sustainedly, the histological findings revealed that SEMS-mediated RFA successfully created the circumferential ablation zone. The RFA-treated groups were observed with decreased markers of cellular proliferation and collagen deposition compared to the control group. Furthermore, IHC findings in the RFA-treated groups revealed increased heat-induced necrosis along with Hsp70 expression indicating that SEMS-mediated RFA could effectively treat stent-induced tissue hyperplasia by coagulative necrosis caused by RFA.

The stented rat gastric outlet is a well-established inexpensive animal model that provides the mechanisms of in-stent restenosis (Park et al., 2020). In our study, the placed the SEMS in the rat gastric outlet with sutures that helped prevent its migration successfully generated stent-induced granulation tissue formation which resulted from mechanical injuries of the gastric outlet tissues within 4 weeks after stent placement. RFA was performed at 4 weeks to evaluate the therapeutic effects of in-stent restenosis after granulation tissue formation had already occurred. It is well known that in-stent restenosis caused by stent-induced tissue hyperplasia of non-vascular luminal organs, such as the esophagus, urethra, biliary duct, and gastric outlet, occurs as an excessive proliferative response within 4 weeks after mechanical injury due to stent placement (Jun et al., 2017; Kim et al., 2018; Park et al., 2018; Park et al., 2020; Jeon et al., 2022; Park et al., 2022). Our current findings support the fact that the local SEMS-mediated RFA is an effective therapeutic option for the treatment of stent-induced granulation tissue formation in the rat GOO model.

In our study, the effects of repeated RFA at 4 and 8 weeks were evaluated. Furthermore, the results of single RFA at 4 weeks and housed for 8 weeks were obtained to evaluate the rebound effect after the discontinuation of RFA. The single RFA-treated group at 4 weeks showed increased cellular necrosis with a heat shock response along the intestinal glands. Stent patency in the RFA-treated groups was significantly increased compared with the control group. However, further follow-up for 4 weeks after RFA confirmed the slightly recurrence of tissue hyperplasia secondary to mechanical injuries of the SEMS and heat-induced fibrotic changes without statistical significance. The stent patency of the repeated RFA-treated group at 4 and 8 weeks was successfully maintained for 8 weeks compared with the single RFA-treated group. The rats in group D that received repeated RFA at 4 and 8 weeks after SEMS placement had a significant decrease in granulation tissue formation at 8 weeks compared to the animals in group C that received a single RFA at 4 weeks. The SEMS-mediated RFA every 4 weeks or periodic RFA could effectively treat stent-induced tissue hyperplasia after SEMS placement. Further long-term follow-up studies are required to determine whether it performs well.

Several investigations have reported that local heat treatment successfully treated stent-induced tissue hyperplasia at 40–65°C (Landsberg et al., 2009; Song et al., 2016; Garanina et al., 2020; Cho et al., 2021). Since Garanina et al. (2020) reported that localized hyperthermia can induce cell necrosis of the entire cell population at temperatures of 58°C–60°C, 60°C was chosen to maximize the RFA effects with cell death in this study. Furthermore, optimization of the RFA parameter was required because the current study was conducted in a rat GOO model, which is anatomically different from those of humans and previous studies (Park et al., 2022). Therefore, the ablation range was analyzed at 60, 180, 240, and 300 s in the porcine liver. However, the ablated liver at 60 s did not archive a uniform ablation zone; therefore, RFA at 60 s was excluded for further evaluation. The SEMS-mediated RFA optimization in the exposed rat gastric outlet was conducted in 120, 180, 240, and 300 s to confirm the presence of perforation or any RFA-related complications. Significant tissue changes with erythema were observed from 120 s onward. After 240 s, severe burning tissues were observed throughout the entire tissue around the implanted SEMS. Histological findings confirmed the insufficient heat shock reaction where only the villi were ablated under 120 s RFA. Overheated damages around the submucosa and adjacent organs were, however, observed over 240 s RFA. Therefore, the appropriate RF energy for the rat GOO model was set at 180 s to maximize RFA effects while minimizing damages to adjacent organs.

Several SEMS-mediated RFA studies were conducted to validate the feasibility and efficacy, and RFA using the SEMS could achieve uniform, circumferential, and transmural coagulative necrosis (Goldberg et al., 1997; Byun et al., 2003; Antunes et al., 2010; Won et al., 2022). However, these studies were conducted only in extracted liver or normal animal models. The rat GOO model was used in this study by inserting the SEMS into the normal rat gastric outlet and inflicting mechanical injury on the tissue (Diegelmann and Evans, 2004; Li et al., 2007). All animals were successfully formatted for moderate-to-severe GOO by granulation tissue formation, in which actual proliferation reduction could be observed in the RFA-treated groups. In the previous studies, the RF delivery system was contacted in the middle portion of a stent to obtain the uniform ablation range; however, tissue ablation beyond the ends of the stent margin (dumbbell shape) and non-uniformed tapering coagulation necrosis in longer stents (>50 mm) were observed (Goldberg et al., 1997; Bernard et al., 2014). In our study, simply grasping the proximal end of the implanted short stent (10 mm) provided the circumferential ablation zone in the porcine liver and uniform coagulative necrosis in the rat GOO model without RFA-related complication. Therefore, the optimized RFA parameter might be effective and safe to create uniform circumferential ablation ranges in the rat GOO model.

The implanted SEMS-mediated RFA using an endoscopic submucosal dissection knife could simply grasp a novel therapeutic strategy for high-grade in-stent restenosis. The monopolar SB Knife was easily delivered through the working channel of endoscopy and then simply graphed the implanted SEMS to perform RFA under endoscopic visualization. Although additional studies are needed for clinical application, the proposed new method has a great potential to treat tumor in/overgrowth and/or granulation tissue formation through the stent meshes after stent placement and to expand indications to other non-vascular luminal organs such as the colon, esophagus, urethra, and airway (Goldberg et al., 1997).

The current study had several limitations. First, a few representative markers of stent-induced tissue hyperplasia and hyperthermia-related marker of RFA were evaluated in this study. Second, the pathological mechanisms that occurred after a stent placement in this study may not always be consistent between humans and our findings. Third, the lack of a temperature and impedance feedback system may hamper the usability of effective RFA. Finally, an additional long-term follow-up study after repeated SEMS-mediated RFA is necessary to evaluate the sustained effectiveness of repeated or periodic treatment.

In conclusion, stent-induced tissue hyperplasia was significantly evident in the stented rat gastric outlet at 4 weeks after stent placement. The uniform circumferential ablation zone was achieved by simply grasping the proximal end of an implanted SEMS to deliver RFA energy to tissue. The implanted SEMS-mediated RFA successfully managed stent-induced tissue hyperplasia and repeated or periodic RFA seems to be more effective to treat in-stent restenosis in the rat GOO model. Although additional studies were needed to verify its safety and efficacy in a large animal model, the implanted SEMS-mediated RFA has therapeutic potential for benign and/or malignant tissue growth through wire meshes after stent placement in non-vascular luminal organs.

## Data Availability

The datasets presented in this article are not readily available due to its proprietary nature, and neither the data nor the source of the data can be made available publicly, but the data presented in this study are available on request from the corresponding author. Requests to access the datasets should be directed to J-HP, jhparkz@amc.seoul.kr.
