# Supplementary material for: Radiofrequency ablation via an implanted self-expandable metallic stent to treat in-stent restenosis in a rat gastric outlet obstruction model
Source: Front Bioeng Biotechnol. 2023 Sep 6;11:1244569. doi: 10.3389/fbioe.2023.1244569 (PMC10516293; doi:10.3389/fbioe.2023.1244569)
Supplement: Supplementary file 1 [file DataSheet1.docx]

SUPPLEMENTARY MATERIALS

**Radiofrequency Ablation via Implanted Self-expandable Metallic Stent to Treat In-stent Restenosis in a Rat Gastric Outlet Obstruction Model**

*Dong-Sung Won^1†^,* *Yubeen Park^1,2†^, Chu Hui Zeng^1^, Dae Sung Ryu^1,2^, Ji Won Kim^1,2^, Jeon Min Kang^1^, Song Hee Kim^1,2^, Hyung-Sik Kim^3^, Sang Soo Lee^2^, Jung-Hoon Park^1^*

*^1^Biomedical Engineering Research Center, Asan Institute for Life Sciences, Asan Medical Center, 88 Olympic-ro 43-gil, Songpa-gu, Seoul 05505, Republic of Korea*

*^2^Department of Gastroenterology, Asan Medical Center, University of Ulsan College of Medicine, 88 Olympic-ro 43-gil, Songpa-gu, Seoul 05505, Republic of Korea*

*^3^Departemnt of Mechatronics Engineering, School of ICT Convergence Engineering, College of Science & Technology, Konkuk University, Chungju, Republic of Korea*

^*^D.-S.W. and Y.P. contributed equally to this work and are the co-first authors.

**Correspondence:**

Sang Soo Lee, M.D. Ph.D.

Department of Gastroenterology, Asan Medical Center, University of Ulsan College of Medicine, 88 Olympic-ro 43-gil, Songpa-gu, Seoul 05505, Republic of Korea

Tel: 82-2-3010-3187 Fax: 82-2-476-0090

E-mail: [ssleedr@amc.seoul.kr](mailto:ssleedr@amc.seoul.kr)

Jung-Hoon Park, Ph.D.

Biomedical Engineering Research Center, Asan Institute for Life Sciences, Asan Medical Center, 88 Olympic-ro 43-gil, Songpa-gu, Seoul 05505, Republic of Korea

Tel: 82-2-3010-4123 Fax: 82-2-476-0090

E-mail: [jhparkz@amc.seoul.kr](mailto:jhparkz@amc.seoul.kr)


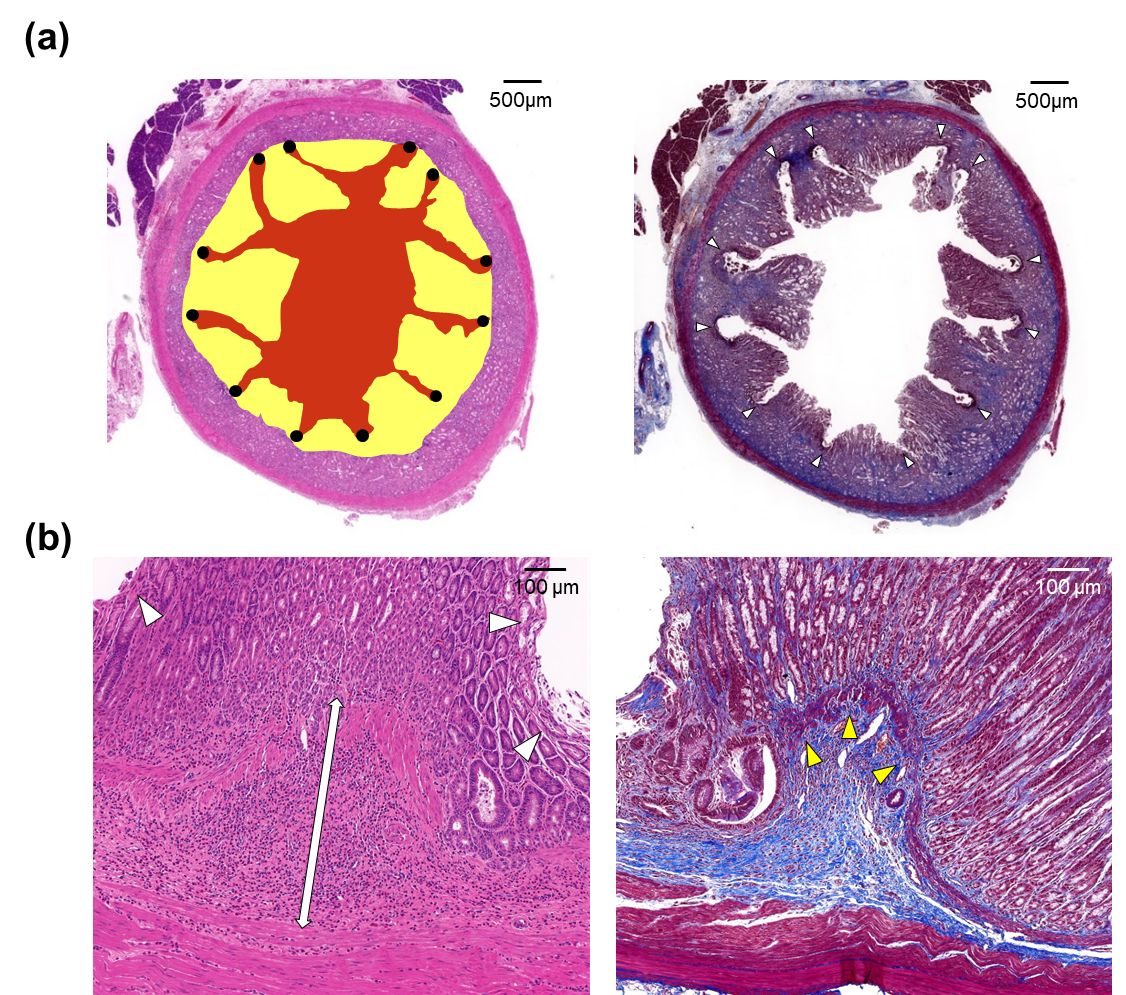


**Supplementary Figure 1.** Analysis methods of histologic examinations for the stented gastric outlet lumen. **(a)** The percentage of tissue hyperplasia area was calculated as 100 × [1 − (stenotic area of stent/original area of stent)]: stent struts (*black dot* and *white arrowheads*), stenotic area (*red*), original area (*yellow*). **(b)** The thickness of the submucosal fibrosis (*double arrow*) from strut (*white arrowheads*) to the submucosal layers and degree of collagen deposition (*yellow arrowheads*) were analyzed in the high magnified histological images (×18.5).


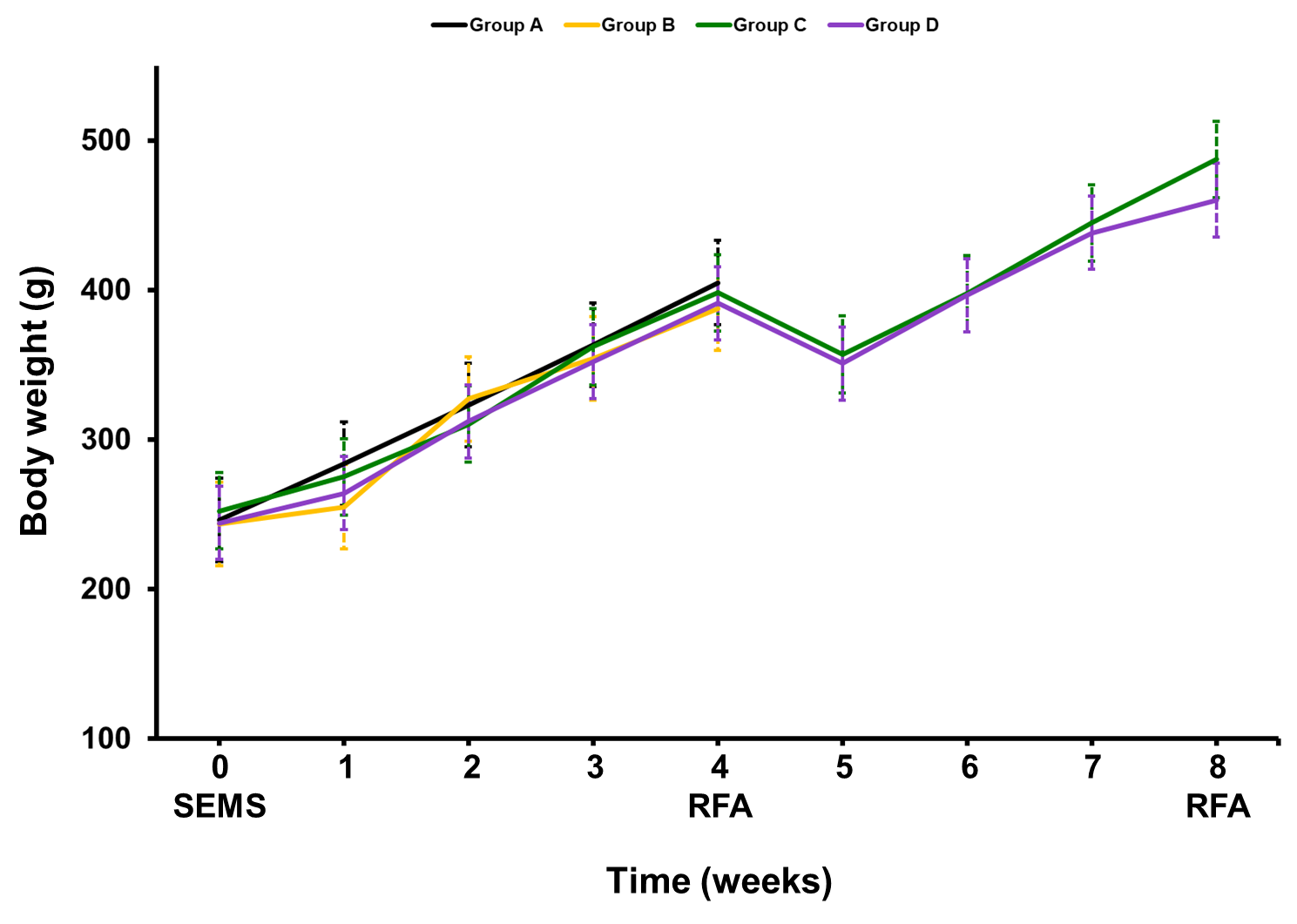


**Supplementary Figure 2.** Change in body weight during the study.
